# MRI imaging manifestations of severe toxic encephalopathy induced by excessive metoclopramide and multiple drug overdose: a case report

**DOI:** 10.3389/fphar.2025.1683132

**Published:** 2025-09-24

**Authors:** Pengfei Liang, Yuncheng Tang, Xiaolin Zhang, Li Zhang, Juan Zhao, Haowen Tang, Zeyu Wu, Qiming Fang, Zhicheng Fang, Zhiwen Zhao, Xuefang Liu

**Affiliations:** ^1^ Department of Emergency Medicine, Taihe Hospital, Hubei University of Medicine, Shiyan, Hubei, China; ^2^ Hubei Provincial Clinical Research Center for Pneumoconiosis and Poisoning, Wuhan, Hubei, China

**Keywords:** metoclopramide, drug overdose, toxic encephalopathy, magnetic resonance imaging, hemoperfusion

## Abstract

**Objective:**

To investigate the clinical and MRI features of severe central nervous system injury caused by an extremely high dose of metoclopramide in combination with multiple drug overdoses, and to summarize treatment strategies and prognosis.

**Methods:**

We report the clinical course of acute poisoning in a 36-year-old woman following a single oral intake of approximately 350 mg metoclopramide combined with adrenolizone and coenzyme Q10. Clinical manifestations, laboratory findings, imaging results (CT and MRI), and treatment interventions after admission were collected and analyzed. The possible pathological mechanisms were explored in conjunction with previous literature.

**Results:**

Upon admission, the patient exhibited acute mental and behavioral disturbances, extrapyramidal symptoms, and impaired consciousness. Initial CT scans revealed no abnormalities. On the fourth day after admission, MRI demonstrated symmetrical patchy hyperintensities on T2WI/T2-FLAIR in the corpus callosum, bilateral corona radiata, and centrum semiovale white matter, along with hyperintensities on DWI and corresponding low ADC values, indicating cytotoxic edema consistent with toxic-metabolic encephalopathy. Following comprehensive multidisciplinary management-including early gastrointestinal decontamination, activated charcoal adsorption, HA380 hemoperfusion, hyperbaric oxygen therapy, neuroprotection, and symptomatic support-the patient was discharged on day 12. At the 3-month follow-up, no neurological sequelae were observed.

**Conclusion:**

Extremely high-dose metoclopramide, particularly in combination with other drugs, can cause a characteristic symmetrical white matter injury pattern of toxic encephalopathy, with MRI findings offering high diagnostic value. Early recognition, prompt gastrointestinal decontamination, blood purification, and multi-target neuroprotective therapy can markedly improve prognosis.

## 1 Introduction

Metoclopramide is a commonly used prokinetic agent widely employed in the management of gastrointestinal dysfunction and in controlling symptoms such as nausea and vomiting (Vimonsuntirungsri et al., 2024). Its primary mechanism of action involves antagonism of central and peripheral dopamine D_2_ receptors, partial activation of 5-hydroxytryptamine (5-HT_4_) receptors, and antagonism of 5-HT_3_ receptors, thereby regulating gastrointestinal motility (McCallum et al., 2024). However, metoclopramide can cross the blood–brain barrier and is transported centrally as a substrate of P-glycoprotein (P-gp) (Breuil et al., 2025). When administered at high doses or over prolonged periods, it may induce extrapyramidal reactions, psychiatric and behavioral disturbances, and toxic encephalopathy. Repeated use can result in death from torsades de pointes ventricular tachycardia (Watanabe et al., 2022). Additionally, it may cause atrial fibrillation (Saleh et al., 2020), serotonin syndrome (Meegada et al., 2019), and persistent hypotension (Nguyen and Petzel Gimbar, 2013). Nevertheless, poisoning from extremely large doses is exceedingly rare, often presenting with complex clinical manifestations, rapid progression, and potentially fatal outcomes.

In recent years, neuroimaging-particularly MRI-has played a crucial role in diagnosing drug-induced toxic encephalopathy (Parbhu et al., 2009; Shang et al., 2025). Toxic-metabolic encephalopathy typically presents as symmetrical signal abnormalities in the corpus callosum and deep white matter, which are valuable for identifying the etiology and assessing prognosis. However, reports on the MRI features, comprehensive treatment approaches, and prognosis of metoclopramide-related multi-drug massive overdose remain scarce.

This article reports the case of a female patient who ingested approximately 350 mg metoclopramide together with adrenolizone and coenzyme Q10 in a single episode. We provide a detailed account of her clinical presentation, MRI findings, and treatment course, and discuss the underlying pathological mechanisms and key clinical management considerations in light of the literature, aiming to offer a reference for the diagnosis and treatment of such rare cases.

## 2 Diagnosis and treatment process

The patient was a 36-year-old woman with no significant past medical history. Approximately 9 h prior to admission, she developed acute mental and behavioral disturbances, characterized by sudden emotional outbursts without apparent cause, episodes of crying, facial twitching, slurred speech, tongue protrusion, voluntary undressing in public, and associated vomiting and hypersalivation. Subsequently, she became progressively apathetic and unable to communicate verbally. Multiple empty medication packages (metoclopramide, adrenolizone, and coenzyme Q10) were found at the scene.

According to her family, the patient had ingested approximately 70 tablets of metoclopramide (5 mg each), 10 tablets of adrenolizone (2.5 mg each), and 10 tablets of coenzyme Q10 (10 mg each) in a single episode, with suicidal intent. Within the 9 h following ingestion, she did not receive any emergency interventions such as gastric lavage or induced emesis, and no other medical records were available.

Physical examination on admission: Body temperature 36.3 °C, pulse 66 beats/min, respiratory rate 18 breaths/min, blood pressure 107/73 mmHg. The patient was delirious, with blunted responsiveness, slurred speech, and impaired communication. Pupils were equal and round, approximately 3 mm in diameter, with preserved light reflexes. Upward gaze deviation and abnormal tongue protrusion were observed. Increased neck muscle tone was noted. The extremities were symmetric; withdrawal from painful stimuli was present, with muscle strength estimated at grade IV, and overall normal limb muscle tone. Deep tendon reflexes were present and symmetric, with no pathological reflexes elicited. Sensory and cerebellar examinations could not be performed due to impaired consciousness. No other focal neurological signs were detected. Glasgow Coma Scale (GCS) score was 10 (E3, V2, M5).

Laboratory findings: Arterial blood gas: pH 7.468, PaCO_2_ 30.7 mmHg, PaO_2_ 109 mmHg, Na^+^ 137 mmol/L, K^+^ 4.03 mmol/L, Ca^2+^ 1.013 mmol/L, glucose 6.0 mmol/L, BE −0.91 mmol/L, HCO_3_^−^ 21.8 mmol/L, COHb 2.0%. Complete blood count: RBC 4.12 × 10^12^/L, WBC 9.52 × 10^9^/L, PLT 322 × 10^9^/L. Liver and renal function: ALT 10.1 U/L, AST 14.2 U/L, total bilirubin 15.98 μmol/L, urea 3.32 mmol/L, creatinine 50.60 μmol/L, uric acid 303.5 μmol/L. Creatine kinase: CK 51.1 U/L, CK-MB 8.5 U/L. Coagulation profile: PTA 87.00%, PT 11.90 s, INR 1.11, APTT 30.60 s, fibrinogen 3.39 g/L, TT 15.20 s.

Electrocardiogram (ECG) Findings: The ECG shows a sinus rhythm with a normal overall pattern. Both the atrial and ventricular rates are 71 beats per minute. The P-wave duration is 90 ms, the QT interval is 388 ms, and the corrected QT interval (QTc) is 422 ms. The PR interval measures 143 ms, and the QRS duration is 73 ms. The RV5/SV1 amplitude ratio is 0.802/1.167 mV, and the RV1/SV5 amplitude ratio is 0.09/0.191 mV.

**FIGURE 1:**
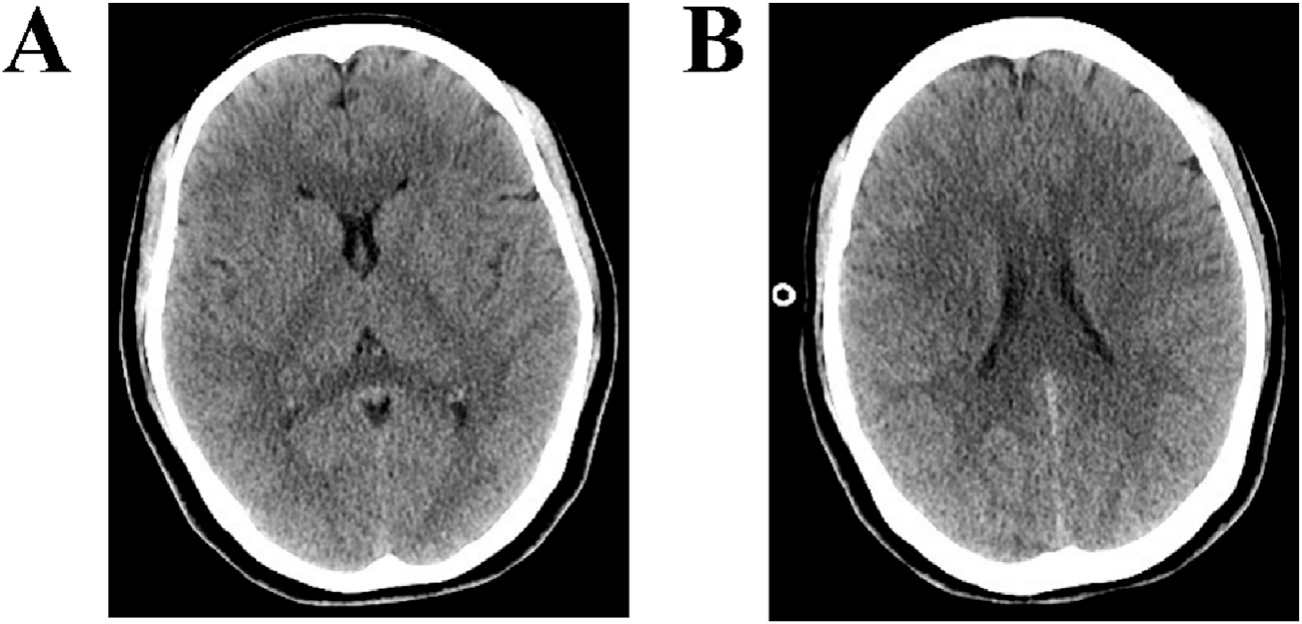
CT findings of the brain. **(A,B)** CT of the brain shows mild narrowing of the cerebral sulci, with mild hypodensity in the white matter, and the changes were symmetrically distributed.

Imaging: Non-contrast brain CT on the day of admission (Figures 1A,B) showed mild narrowing of the cerebral sulci, with mild hypodensity in the white matter, and the changes were symmetrically distributed.

Immediately upon admission, intravenous access was established, and a nasogastric tube was inserted for repeated gastrointestinal decompression. Activated charcoal was administered via the nasogastric route to adsorb residual drugs, and a liquid glycerin enema was performed to facilitate excretion. Oral benzhexol was prescribed to alleviate extrapyramidal reactions, while intermittent intravenous diazepam or midazolam was given for sedation, anticonvulsant purposes, and relief of dystonia. Omeprazole was administered to suppress gastric acid secretion, and bismuth potassium citrate was used to protect the gastric mucosa. Active fluid resuscitation was initiated, combined with diuretics to promote toxin elimination. Water, electrolyte, and acid-base balance were closely monitored. Pharmacokinetic testing was recommended to the patient’s family; however, they declined.

Given the patient’s intake of multiple oral medications in large doses and the presence of significant extrapyramidal symptoms, along with the indication for blood purification, hemoperfusion was selected as the intervention. This treatment aimed to mitigate toxic effects by removing harmful substances from the blood. The hemoperfusion device used was the JianFan HA380 model, with a flow rate of 200 mL/min and a duration of 4 h. Prior to the hemoperfusion, the patient’s GCS score was 10 (E3, V2, M5), with an on-machine blood pressure of 108/66 mmHg and a pulse of 59 beats per minute. During the procedure, the patient’s blood pressure remained around 95/66 mmHg, with an on-machine blood pressure of 99/64 mmHg and a pulse rate of 53 beats per minute. After the hemoperfusion, the GCS score improved to 12 (E4, V3, M5).

On the third day of admission, the patient was conscious with an average mental state, able to follow simple instructions, but with non-fluent speech. Repeat laboratory tests revealed hypoproteinemia (albumin 30.51 g/L), likely related to insufficient intake after poisoning, increased protein catabolism under stress, and altered capillary permeability. Mild anemia (Hb 107 g/L) was also detected, possibly associated with acute inflammatory response, malnutrition, and hemodilution. Dietary adjustments were made to increase the intake of high-quality protein.

**FIGURE 2:**
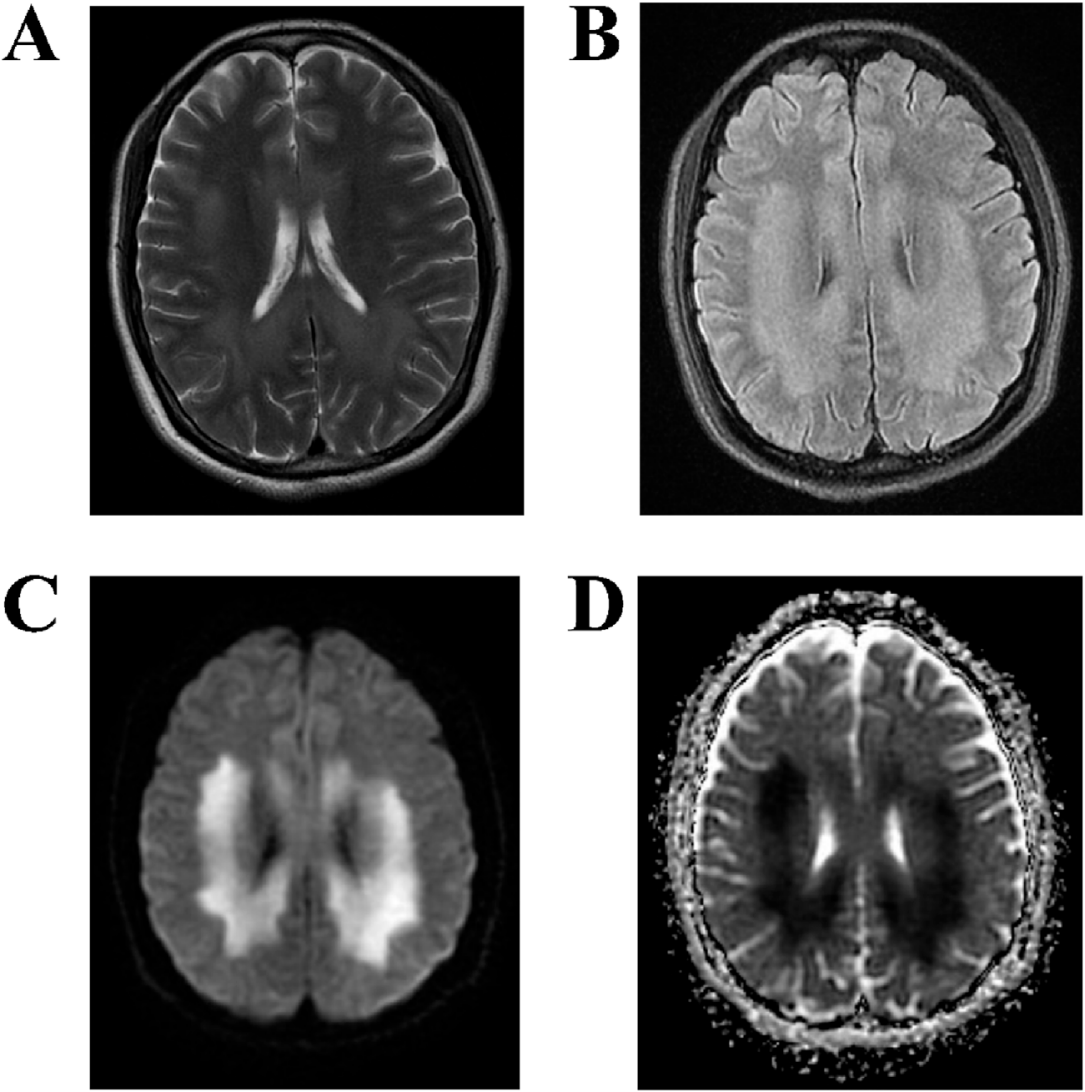
MRI findings of the brain. **(A,B)** T2WI and T2-FLAIR sequences demonstrate symmetrical patchy hyperintensities in the corpus callosum, bilateral corona radiata, and centrum semiovale white matter. **(C,D)** DWI reveals symmetrical patchy hyperintensities in the same areas, with corresponding low signal intensity on the ADC map, indicating restricted diffusion.

MRI examination on the fourth day of admission (Figures 2A–D) revealed the following: T2-weighted imaging (T2WI) (Figure 2A) showed symmetrical patchy hyperintensities in the corpus callosum, bilateral corona radiata, and the white matter of the centrum semiovale. T2-FLAIR (Figure 2B) similarly revealed symmetrical patchy hyperintensities in the same regions. Diffusion-weighted imaging (DWI) (Figure 2C) showed symmetrical patchy hyperintensities at the corresponding sites. The apparent diffusion coefficient (ADC) map (Figure 2D) showed low signal intensity in the same areas, indicating restricted diffusion. These imaging findings are consistent with diffuse white matter injury caused by drug poisoning, affecting the corpus callosum and bilateral centrum semiovale, with a symmetrical distribution pattern characteristic of toxic-metabolic encephalopathy.

A multidisciplinary consultation with the hyperbaric oxygen and neurology departments was conducted immediately, and hyperbaric oxygen therapy was initiated to improve cerebral tissue oxygenation, promote resolution of brain edema, reduce free radical-mediated damage, and facilitate neurological recovery. Concurrently, intravenous citicoline was administered to repair neuronal cell membranes and enhance neurotransmitter synthesis. Mannitol was given for osmotic dehydration to reduce intracranial pressure, and Dexamethasone Sodium Phosphate were used to suppress inflammatory responses and stabilize the blood-brain barrier. In addition, B vitamins, and edaravone were prescribed. Throughout the treatment period, intracranial pressure was closely monitored, and daily neurological examinations were performed to assess muscle strength, tone, and reflexes. As shown in Table 1, the treatment measures received during the hospital stay and their durations.

**TABLE 1:** Treatment strategies and adverse effect monitoring during hospitalization.

Treatment/Medication name	Dosage and administration	Start time (Relative to admission)	Stop time (Relative to admission)	Expected mechanism	Monitoring for adverse effects
Hyperbaric Oxygen Therapy	Hyperbaric oxygen chamber, 1 session per day, 60 min per session	Started on day 4 after admission	Continued for 10 days	Improves cerebral tissue oxygenation, promotes resolution of brain edema, reduces free radical-mediated damage, and facilitates neurological recovery	Daily monitoring of oxygenation status, observation of potential adverse effects
Citicoline	Intravenous injection, 500 mg/day	Started on day 4 after admission	Continued for 7 days	Repairs neuronal cell membranes, enhances neurotransmitter synthesis	Monitoring of liver function, watch for possible allergic reactions
Mannitol	Intravenous injection, 1.5 g/kg/day	Started on day 4 after admission	Continued for 7 days	Osmotic dehydration to reduce intracranial pressure	Daily monitoring of blood pressure, observation of potential electrolyte imbalance
Dexamethasone Sodium Phosphate	Intravenous injection, 20 mg per dose	Started on day 4 after admission	Continued for 7 days	Suppresses inflammatory responses and stabilizes the blood-brain barrier	Monitoring of weight, blood glucose levels, observation of gastrointestinal discomfort or potential long-term side effects
B Vitamins	Intravenous injection, 2g/day	Started on day 4 after admission	Continued for 7 days	Promotes nerve repair and enhances neurotrophic effects	Daily monitoring of liver and kidney function, watch for allergic reactions
Edaravone	Intravenous injection, 30 mg/day	Started on day 4 after admission	Continued for 7 days	Enhances antioxidant capacity and improves neuronal cell damage	Monitoring of liver function, liver protection, and cellular repair

**FIGURE 3:**
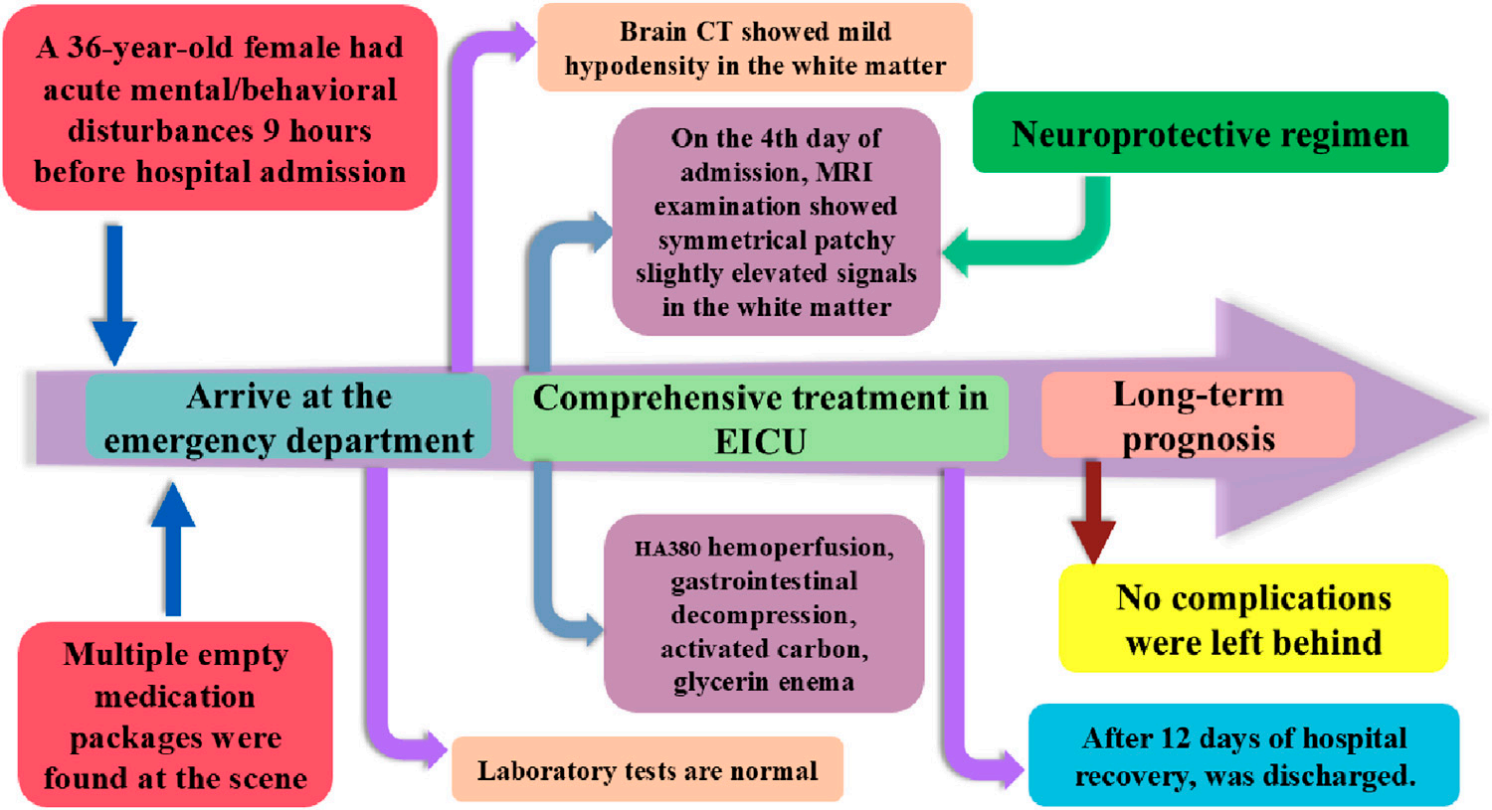
The inpatient treatment and prognosis timeline of the patient.

On the 10th day of admission, the patient was alert, oriented, and hemodynamically stable, able to perform activities of daily living independently, with normal eating and sleeping patterns. Hyperbaric oxygen therapy was discontinued after a total of 10 days. On the 12th day, re-examination revealed no neurological deficits, normal limb strength and coordination, absence of extrapyramidal signs, steady gait, and overall clinical stability. The patient was discharged. At the 3-month follow-up, the patient demonstrated complete physical recovery without any neurological sequelae. As shown in Figure 3, this chart illustrates the inpatient treatment and prognosis timeline of the patient.

## 3 Discussion

Metoclopramide is widely used in the treatment of gastrointestinal disorders and for the control of nausea and vomiting (Vimonsuntirungsri et al., 2024). Its primary pharmacological actions include antagonism of central and peripheral dopamine D_2_ receptors, partial activation of 5-HT_4_ receptors, and antagonism of 5-HT_3_ receptors (McCallum et al., 2024). However, it can induce movement disorders or exacerbate pre-existing extrapyramidal syndromes (Alkhowaiter et al., 2024), with an estimated incidence of approximately 0.2% (Jo et al., 2012). Common adverse effects include akathisia, nystagmus, acute dystonic reactions (metoclopramide-induced acute dystonic reactions, MIADRs) (Fink et al., 2023), and restless legs syndrome (Aljunaid, 2024). In rare cases, it may precipitate a pheochromocytoma crisis and be complicated by acute respiratory distress syndrome (ARDS) (Xie et al., 2023) or neuroleptic malignant syndrome (NMS) (Wittmann et al., 2016). When the daily dose exceeds 30 mg, the risk of MIADRs increases significantly (Malakian et al., 2025). In the present case, the patient ingested approximately 350 mg of metoclopramide in a single episode, together with adrenolizone and coenzyme Q10-far exceeding the safe therapeutic range. This presentation is classified as a massive multi-drug overdose.

Carbazochrome is a capillary hemostatic agent (Alsaied et al., 2025). It works by enhancing the contraction of damaged capillaries and reducing inflammation-induced endothelial permeability, thus helping to maintain microvascular integrity (Sendo et al., 2003). This mechanism plays a key role in minimizing the risk of bleeding. In clinical practice, Carbazochrome generally causes few side effects, and no studies have reported any adverse effects on the central nervous system (CNS) at standard therapeutic doses.

In this case, MRI demonstrated symmetrical patchy hyperintensities in the corpus callosum, bilateral corona radiata, and the central white matter of the centrum semiovale. DWI revealed corresponding hyperintensities with low ADC values, consistent with the cytotoxic edema pattern characteristic of toxic–metabolic encephalopathy. Compared with other toxic agents, endosulfan poisoning can rapidly induce central nervous system excitation and status epilepticus, with MRI findings suggestive of diffuse cerebral edema (Parbhu et al., 2009). Exposure to methyl iodide may produce symmetrical patchy signal abnormalities (Liu et al., 2025). Occupational poisoning from 1,2-dichloroethane often involves the cerebellar dentate nucleus, basal ganglia, and bilateral cerebral white matter, and the extent of imaging abnormalities does not always correlate with clinical severity (Wang et al., 2025). In methanol poisoning, acute symmetric ischemic changes with cytotoxic edema can be seen in the basal ganglia and bilateral optic nerves (Balodis et al., 2024). In addition, poisoning by substances such as potassium cyanide (Messing, 1991), Endosulfan (Parbhu et al., 2009), Carbon monoxide (Aoshima et al., 2021), dimethylamine borane (Yu et al., 2022), and Hydrogen sulfide (Genjiafu et al., 2023) often results in symmetrical involvement of the corpus callosum and deep white matter. A history of excessive exposure to these substances can help suggest a diagnosis.

The management of metoclopramide overdose should be guided by the drug’s toxicological profile and implemented through a multi-dimensional, collaborative approach. Given that metoclopramide is highly lipophilic, capable of crossing the blood-brain barrier (BBB), and a substrate of P-gp, rapid reduction of its systemic and cerebral burden is critical for improving prognosis. During the acute phase, prompt gastrointestinal decontamination is essential. Early gastric lavage and repeated administration of activated charcoal upon admission can reduce ongoing absorption. In patients who do not receive timely intervention, gastric decompression via nasogastric tube followed by nasogastric administration of activated charcoal can be performed to adsorb residual drug. In the present case, such decontamination procedures were completed approximately 9 h after ingestion, providing a foundation for subsequent therapeutic measures.

Secondly, given that metoclopramide acts on central dopamine receptors, patients frequently develop severe extrapyramidal reactions. Therefore, agents such as benzhexol should be administered during treatment to alleviate these symptoms. In addition, if severe seizures or psychiatric disturbances occur, prompt administration of intravenous diazepam, midazolam, or similar agents is warranted for sedation and anticonvulsant therapy.

Furthermore, a multi-target neuroprotective regimen may be employed, combining citicoline to repair neuronal cell membranes, edaravone to scavenge free radicals, and mannitol to reduce intracranial pressure. Hyperbaric oxygen therapy can enhance cerebral oxygenation, reduce myelin edema, and facilitate the repair of white matter injury. In the present case, a cumulative 10-day course of such interventions markedly accelerated neurological recovery.

In terms of systemic support, hemodynamic status should be closely monitored, fluid resuscitation should be actively implemented, and diuresis should be employed when necessary to facilitate toxin clearance. For ultra-high-dose exposures, blood purification represents a key therapeutic intervention. Metoclopramide has a molecular weight of 299.8 g/mol and a plasma protein binding rate of approximately 30% (Wishart et al., 2018), fulfilling the criteria for hemoperfusion. In particular, HA380 resin possesses strong adsorption capacity for lipophilic toxins and can rapidly reduce plasma drug concentrations (Li et al., 2025). In the present case, a 4-h hemoperfusion session was initiated within 1 h of admission, effectively mitigating ongoing central nervous system injury caused by the toxin. It should be noted that the family’s refusal to consent to drug metabolism and toxicology testing limited the ability to quantitatively assess the dose-response relationship of exposure and to optimize individualized detoxification strategies.

## 4 Conclusion

Ultra-high-dose metoclopramide poisoning can result in severe central nervous system injury, presenting with acute psychiatric and behavioral disturbances, extrapyramidal reactions, and toxic encephalopathy. MRI typically demonstrates symmetrical white matter injury, which serves as an important diagnostic indicator. Early gastrointestinal decontamination, activated charcoal adsorption, and blood purification can markedly reduce the toxic burden and improve prognosis. In the present case, the patient was followed up for 3 months after standardized treatment and exhibited no sequelae, underscoring that early recognition and multidisciplinary collaboration are critical to optimizing outcomes in toxic encephalopathy.

## Data Availability

The original contributions presented in the study are included in the article/supplementary material, further inquiries can be directed to the corresponding authors.
